# Using Modeling All Alternatives to explore 55% decarbonization scenarios of the European electricity sector

**DOI:** 10.1016/j.isci.2023.106677

**Published:** 2023-04-18

**Authors:** Tim T. Pedersen, Mikael Skou Andersen, Marta Victoria, Gorm B. Andresen

**Affiliations:** 1 Department of Mechanical and Production Engineering, Aarhus University, Aarhus, Denmark; 2 Department of Environmental Science - Environmental Social Science and Geography, Aarhus University, Aarhus, Denmark; 3 ICLIMATE Interdisciplinary Centre for Climate Change, Aarhus University, Aarhus, Denmark

**Keywords:** Global carbon cycle, Energy management

## Abstract

Climate change mitigation is a global challenge that, however, needs to be resolved by national-level authorities, resembling a “tragedy of the commons”. This paradox is reflected at the European scale, as climate commitments are made by the EU collectively, but implementation is the responsibility of individual Member States. Here, we investigate a suite of near-optimal effort-sharing scenarios where the European electricity sector is decarbonized between 55% and 75% relative to 1990, in line with 2030 ambitions. To this end, we use a brownfield electricity system optimization model in combination with the Modeling All Alternatives methodology. Results show that only very particular effort-sharing schemes are able to reach the theoretical minimum system cost. In most cases, an additional cost of at least 5% is incurred. Results reveal large inequalities in the efforts required to decarbonize national electricity sectors.

## Highlights

• The realized energy system is likely to be 5% more expensive than the theoretical optimum
• The majority of scenarios decarbonize the electricity sector beyond 62% by 2030
• The average cost of abating emissions varies between 0 and 35€/t CO_2_ among nations
• Germany’s emission target has significant impact on central European power prices

## Graphical abstract

**Figure:**
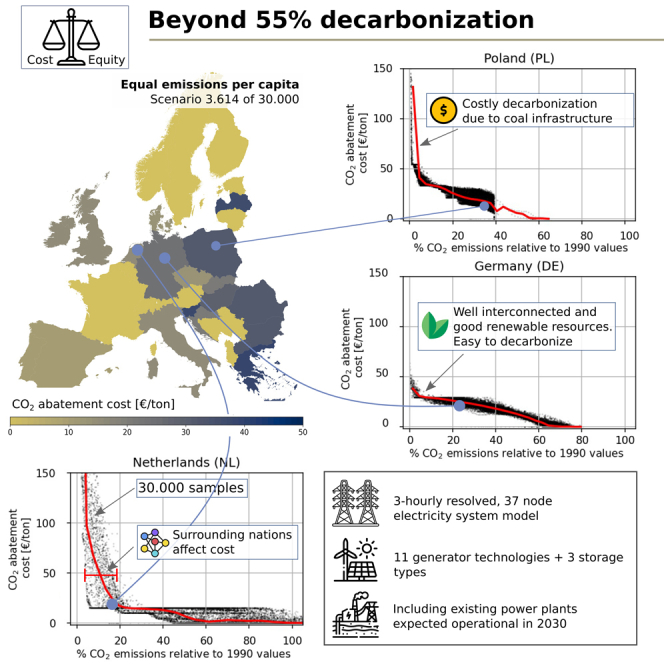

## Introduction

One of the most crucial aspects of climate change mitigation is how to overcome “the tragedy of the commons”.^1^ Climate change is a global problem that cannot be managed without deliberate action from all individual countries on Earth. Agreements to reduce emissions have historically been made as multi-lateral agreements, e.g., global agreements such as the Kyoto Protocol and the Paris Agreement, or lately the European Green Deal.^2^ The implementation of regulations to reduce emissions is, however, to be done at a national level. As strict national targets for emissions reductions are likely to entail a loss in welfare in the short term, the inclination of most countries so far has been to opt for the least ambitious emission target that satisfies their commitment to global agreements.^3^ This can be observed today where the sum of nationally determined contributions, is far from sufficient to meet the targets of the Paris Agreement.^4^ In other words, nations are maximizing their national welfare, but in doing so the common resource, our remaining CO_2_ budget, is exhausted prematurely. This behavior will lead to a decrease in global welfare in the long run, as climate change eventually will entail much higher costs, than what is gained from presently uncurbed emissions.^5^

Harding argues that there exist no technical solutions that can be found to solve such a problem of the commons, other than by dividing up the common resource.^6^ The finite resource—that is our atmosphere’s CO_2_ carrying capability—can be divided through the implementation of a cap-and-trade policy. Setting a quota on emissions that in turn will allow emission rights to be traded in a market with a carbon price should lead to efficient decarbonization of our society,^1^ but at what cost? Bauer et al.^7^ find that huge international financial transfers are required to ensure the justice of the transition when governed by a cap-and-trade system. This significant but unresolved equity issue is replicated at the regional level in Europe, where climate commitments are made by the European Union (EU), but implementation and governance rest with individual countries. In the European Union, there is now a commitment to aim for a just transition as stated in the Green Deal.^2^ Financial support will be provided by the Just Transition Mechanism to those jurisdictions where the socioeconomic impact of the transition will be highest.^8^ The quality of a just transition is, however, not easily measured and has, in recent years, become a topic of much debate.^9^^,^^10^

**Figure 1:**
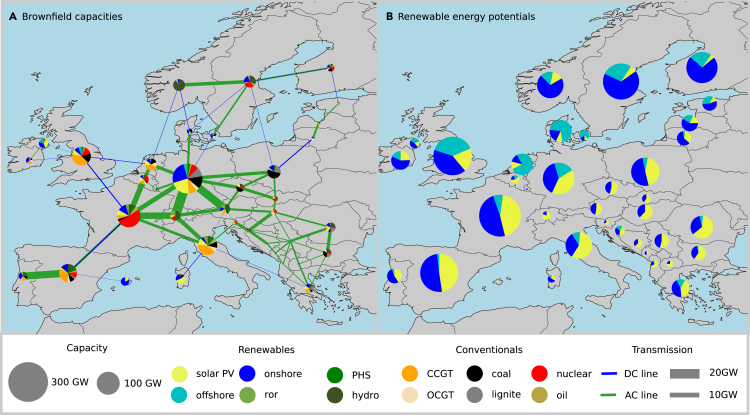
Existing generator capacity and renewable potentials (A) Currently installed technology capacity that is expected to be in operation in 2030. Capacities represent maximum electricity generation. (B) Effective renewable energy potentials, calculated as the maximum geographical potential times the local capacity factor for the given technology. Wind turbine capacity is specified as offshore and onshore. Hydro-power is separated as either run-of-river (ROR), pumped-hydro-storage (PHS), or hydro. Two types of gas turbines are included: closed-cycle-gas-turbines (CCGT) and open-cycle-gas turbines (OCGT). Transmission capacities are indicated either as high-voltage AC or DC transmission lines. The majority of countries have both brownfield and renewable potentials to cover several times their annual electricity demand.

The green transformation of Europe’s domestic energy supply is challenged by several factors of technical, economic, and political nature: the access to renewable resources,^11^ power plants currently in operation, and availability of international transmission connections.^12^ Figure 1A shows the power and transmission capacity installed today and expected to remain in operation by 2030. It is clear, that the preconditions for rapid decarbonization are very diverse, with some countries relying heavily on coal and oil, while others have a large share of renewable power generation. Figure 1B shows the potential for renewables per Member State. Renewable potentials are calculated as the geographical potential for renewable capacity multiplied by the average national capacity factor for the given renewable resource. It is evident that the renewable potentials are unrelated to the nation’s population size and local demand. Given the diverse preconditions for a green transition in the European electricity supply, varied costs associated with the transition must be expected. Ensuring equity in the transition requires the identification of nations where the green transition comes at a higher cost and nations where only minor political incentives are required.

A uniform price on CO_2_ emissions is considered the most efficient way to achieve emissions reductions.^13^ However, with a uniform CO_2_ price, the fairness of the transformation cannot be ensured. The cost-efficient solution obtained with a uniform CO_2_ price is likely to favor early decarbonization in regions where renewable resources are favorable, resulting in a skewed transformation.^14^ If equal effort sharing is to be ensured under a uniform CO_2_ price, financial transfers are required as the cost of decarbonization varies between nations.^7^ Agreeing upon what equal sharing of effort looks like is inherently difficult as it is not a question about costs but rather one of ethics.^15^ Zhou and Wang^16^ identify a range of effort-sharing schemes, based on different principles, such as sovereignty, egalitarianism, efficiency, horizontal equity, vertical equity, and polluter pays. Other studies combine these principles to create more complex effort-sharing schemes, such as the Model of Climate Justice per capita,^17^ where historical emissions along with population growth are considered. Markowitz notes that we are ill-equipped to decide, given the complexity of the problem, and our complicity in causing it.^18^

Several studies exist where the equity efficiency trade-off occurring in the transition of our energy supply has been conducted. Bauer et al.^7^ studied the trade-off to be made between economic efficiency and sovereignty in a global context using a multi-objective approach combined with an integrated assessment model. Their findings indicate a highly non-linear trade-off between efficiency and sovereignty, where the intermediate scenarios can secure higher total benefits. Van den Berg et al.^3^ studied the implications of employing several effort-sharing approaches in a global context, finding a large discrepancy between the cost-optimal solution and the effort-sharing principles. Schwenk-Nebbe and co-authors,^19^ consider three different effort-sharing principles for establishing national CO_2_ reduction targets in a European context. In the Efficiency solution, they find that the remaining emissions will be concentrated in a small number of countries where the costs of decarbonization are highest. Allocating emissions after a Sovereignty or Grandfathering principle will distribute the remaining emissions more evenly while implying a spread in-unit abatement costs and higher total system cost. Sasse et al.^20^ study the trade-off between regional equity and efficiency in the case of Switzerland’s energy supply under varying spatial allocation of generator capacity using a Modeling to Generate Alternatives (MGA) method. Their findings show how renewable energy production will be concentrated in regions with favorable renewable resources if no equity measures are employed. An equivalent study for the German electricity sector has been conducted drawing similar conclusions.^21^ A study investigating the impact of technology mix on equity in the European electricity supply has previously been published by some of the Authors of this paper.^22^ In the work by Pedersen et al.^22^ the Modeling All Alternatives (MAA) method is introduced, capable of exploring all near-optimal alternatives within a given slack on system cost.

The majority of existing literature seeks to address the problem of the equity-efficiency trade-off by imposing varying effort-sharing principles. The underlying problem is that (a) the prerequisites for abating emissions vary widely between nations and (b) there exists no single just way of distributing the burden associated with decarbonizing the electricity supply. Existing literature fails in capturing the complex interplay between nations as they rely on small ensembles of scenarios. To understand how the interactions between nations affect individual countries’ ability to decarbonize, new methods are needed. Furthermore, to guide policymakers in their aim of ensuring a just transition, a vigorous exploration of potential pathways must be identified rather than small sets of scenarios. While Sasse et al.^20^ address this issue to some extent by applying an MGA method, the focus of their study is on the effects of changing spatial allocation of generators and not explicitly on effort sharing. Similarly, the study by Pedersen et al.^22^ is using the MAA method building on the principles from MGA, but again focusing on technology allocation rather than effort sharing.

This work aims to address the difficulties in ensuring distributional justice^9^ in the transformation of the European electricity supply by using a model of the European electricity sector in combination with an implementation of the MAA method,^22^ to investigate an unbiased sample of 30.000 near-optimal emission reduction scenarios. Each scenario represents a unique configuration of national emission reduction targets that may arise from multi-lateral political negotiations. This allows us to answer the following research questions: (1) What is the most probable increase in system cost due to economically in-efficient allocation of emission rights? (2) How likely are scenarios with similar specific emissions and electricity prices in all countries? (3) How sensitive is the country-specific abatement cost and electricity price to the emission reduction targets of other countries?

The contribution of this work lies in the thorough exploration of all possible configurations of national emission targets in a European context, uncovering how the interactions between nations affect decarbonization costs. Where previous studies have used scenario-based modeling or MGA methods to study the implications of a small range of effort-sharing schemes, the MAA method applied in this work is capable of uncovering the underlying problem structure by investigating all possible outcomes. This reveals the complex interactions occurring between nations previously not identified. It is possible because the MAA method allows unbiased sampling of the entire near-optimal space, which means that, unlike other MGA methods, the distribution of the identified near-optimal solutions is not sensitive to the specific value of the cut-off threshold. Finally, the implementation of MAA used here represents a significant improvement in the number of dimensions it is capable of considering. Where the previous method presented in^22^ could investigate upwards of 10 dimensions at the time, 33 dimensions are investigated in this work.

The following section explains the detailed study results, followed by the discussion and method limitations. A detailed description of the methods used is given in the STAR Methods.

## Results

**Figure 2:**
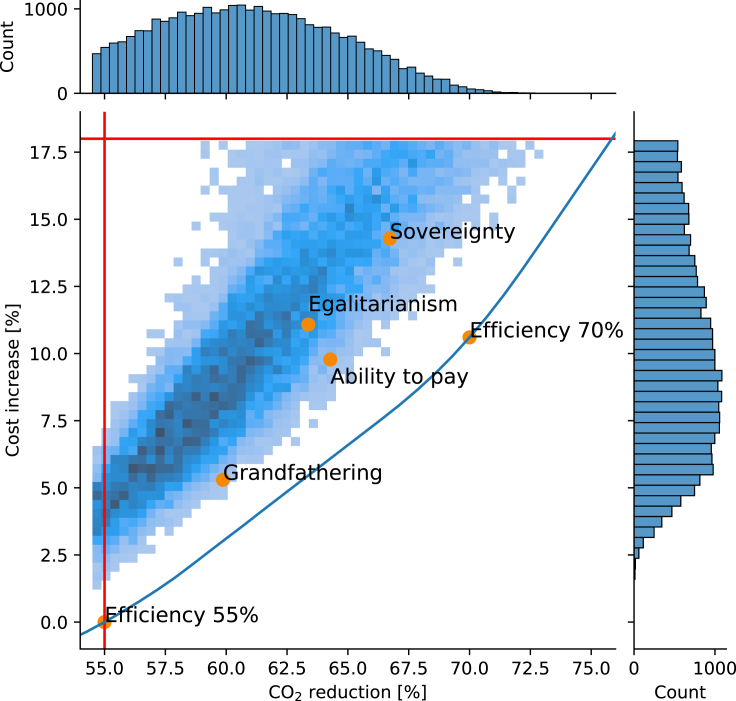
Distribution of possible CO_2_ reduction configurations Histogram showing the CO_2_ reductions relative to 1990 and the associated costs of all feasible configurations of national reduction targets. The minimum required CO_2_ reduction and maximum allowable cost increase is marked with red lines. The blue line marks the Pareto-optimal front of a dual objective optimization procedure using total system cost and joint CO_2_ reduction as the two objective functions. The reduction target scenarios Efficiency 55%, Efficiency 70%, Grandfathering, ability-to-pay, Egalitarianism, and Sovereignty are marked with orange.

Applying the MAA method (implemented using the Adoptive Metropolis Hasting sampler) in combination with the PyPSA-Eur-Sec model^23^ of the European electricity sector, a total of 30.000 near-optimal configurations of national emission targets were drawn. Each scenario has a unique combination of national emission reduction targets. All scenarios are sampled uniformly from the near-optimal solution space of the model. In Figure 2, the resulting realized CO_2_ emissions from all configurations are shown, plotted against their total system costs. Each configuration is required to have an increase in system cost below 18% relative to the cost-optimal solution and a minimum emission reduction of 55% for Europe as a whole. These two cuts are shown as red lines in the figure. Note here that the MAA method ensures that the exact value of the maximum cost increase is not critical as long as the majority of the underlying distribution is captured. Looking at the two marginal distributions on the top and right side of Figure 2 it can be observed that the main body of the two distributions is well within the bounds. The upper bound on emission reduction is in principle 100% which can be achieved if all countries are fully decarbonized. However, the upper limit on system cost is preventing this as decarbonization is associated with increasing system cost. Since the model year is 2030, we considered it reasonable to not spend computational resources on extending the study to higher levels of decarbonization.

Five principle effort-sharing schemes, inspired by Zhou, P. and Wang, M. (2016),^16^ are shown as a reference. The principle schemes and their associated effort-sharing rules are found in Table 1. The Efficiency scheme—representing a uniform CO_2_ price—is implemented with two global emission reduction goals, namely 55% and 70% reductions. The reference scenario with the lowest total system cost is the Efficiency 55% scenario. Further detail on the principle effort sharing schemes is found in the Experimental Procedures. In Figure 2, the Pareto front showing the cost versus CO_2_ reduction trade-off is shown with a blue line. The Pareto optimal front was calculated by continuously decreasing the targeted joint CO_2_ emissions using a uniform CO_2_ price.

**Table 1:** Strategies for CO2 target configurations

Name	Interpretation	Rule
Grandfathering	All nations have equal right to pollute	Distribute emissions proportionally to historical emissions
Sovereignty	All nations have equal right to pollute	Distribute emissions proportionally to energy demand
Efficiency	Maximize global welfare	Distribute emissions to reduce total socio-economic costs
Egalitarianism	All citizens have equal right to pollute	Distribute emissions proportionally to population size
Ability to pay	Nations with higher welfare should take on a larger part of the task	Distribute emissions inversely to GDP per capita

In Figure 2, a gap between the Pareto optimal front and the samples can be observed. Nothing is preventing the sampler from identifying configurations on the Pareto optimal front, it is, however, very unlikely as it requires exact coordination of the nations’ CO_2_ targets. It shows that the most economically efficient (optimal) solution is an extreme scenario that is very hard to obtain without extensive collaboration and agreement between all European countries. This is very unlikely, as countries have individual national targets and agendas. The observation is supported by the marginal distribution of scenario cost on the right-hand side of Figure 2, where it can be observed that very few scenarios with a cost increase below 5% exist. The dark blue regions in Figure 2 represent regions where more scenarios are located. Thus, more scenarios have the properties associated with these regions, e.g., 5–7% higher cost than the optimal solution. Considering the distribution of the joint CO_2_ reductions, at the top of Figure 2, it is clear that the distribution is skewed toward the joint target limit of 55% emission reductions (marked by the vertical red line), but with a peak located around 62% emission reductions. This indicates there are more scenarios achieving low emission reductions rather than over-achieving by a large extent. At the same time, the peak at 62% shows that it is potentially easier to achieve a higher emission reduction than 55% as several nations find it cost-optimal to reduce emissions by 2030.

In Figure 2 all the principle effort-sharing schemes, except Efficiency 55%, are seen to provide a higher CO_2_ reduction than required. This over-performance on emissions reduction is a result of several countries finding it cost-optimal to reduce emissions beyond their assigned national target. Further information on this behavior is available in Figure S1. The Grandfathering and Ability to pay schemes are located relatively close to the Pareto-optimal front, whereas Sovereignty and Egalitarianism are found to be further from it (see Figures 2 and S2). While the distance to the Pareto front can be interpreted as a measure of the economic efficiency of the scheme, the density of the sample distribution around the scheme observed in Figure 2 indicates the distinctiveness of that scheme. A scheme located in a dense region will be achievable with more configurations of the energy system, whereas one located in the sparse regions is likely to require close coordination of national emission targets. Here it is important to note that all scenarios are drawn with equal likelihood. A dominating effort-sharing principle could introduce a bias that would skew the distribution.

**Figure 3:**
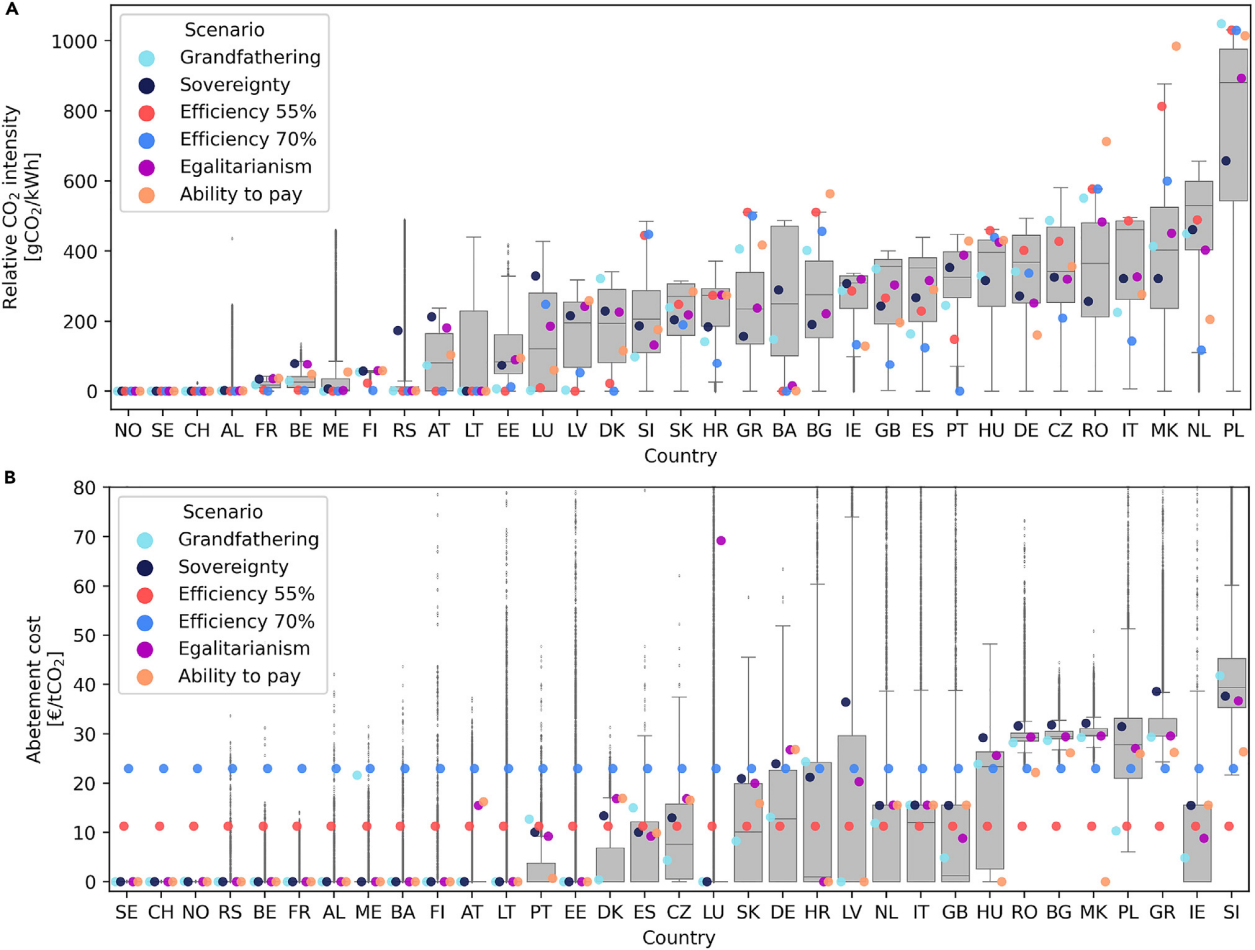
National emission intensity and abatement cost (A) National CO_2_ emissions intensity for all modeled scenarios. Emission intensity is measured as emitted CO_2_ per MWh of produced electricity. (B) CO_2_ abatement cost for all modeled scenarios. Abatement costs are given as the shadow price occurring when imposing national emission constraints. The scenarios of Grandfathering, Sovereignty, Efficiency, Egalitarianism, and ability-to-pay are highlighted.

Figure 3A shows the range of CO_2_ intensity (emissions per kWh) in each of the modeled countries across all modeled scenarios. The average emission intensity for the EU-27 electricity supply was 230 gCO_2_/kWh in 2020 according to European Environment Agency (EEA).^24^ As seen in the figure, all countries have zero emissions in one or more configurations. Countries such as Norway and Sweden have zero emissions under all circumstances. This is not because they are allocated a demanding reduction target, but simply because it is cost-optimal to rely fully on renewable or nuclear energy in the electricity supply of these countries. On the other hand, countries such as Poland and the Netherlands tend to have large emissions intensity in most accepted configurations as scenarios, where these countries have ambitious reduction targets, are very likely to be too expensive and thereby rejected. In 2020, the emission intensity for Poland’s electricity supply was 700 gCO_2_/kWh.^24^ By analyzing the configuration of national reduction targets in the Efficiency approaches, it can be observed that higher than average shares of emissions are allocated to countries that at the outset have high emissions and less than average shares of emissions are allocated to countries with low initial emissions. In other words, the Efficiency schemes favor assigning modest reduction targets to countries that have a hard time reducing emissions and cutting emissions drastically in countries where CO_2_ reduction is easier. This intuitively reduces total system cost but increases inequality. When comparing the Efficiency scenario with 55% and 70% reductions, we can identify countries that are next in line to reduce emissions. Netherlands, Italy, and Portugal are all seen to reduce emissions substantially in the 70% scenario compared to the 55% scenario. Other nations find the emission intensity unchanged even though joint emissions have been reduced.

Imposing a limit on national CO_2_ emissions naturally triggers a shadow price on emissions abatement. In a linear optimization model such as the one used in this paper, the Lagrange/Karush Kuhn-Tucker multiplier of the national CO_2_ constraints serves as a proxy for the national CO_2_ abatement cost. The Lagrange multiplier measures the change in system cost caused by a marginal change in the given constraint. The CO_2_ abatement costs can be interpreted as the policy push—e.g., in the form of subsidies or other political incentives—required to obtain a given national emission target. High abatement cost shows that the electricity supply is hard to decarbonize. CO_2_ abatement costs for all modeled configurations are shown in the top panel of Figure 3B. An abatement cost only occurs when the national emission reduction constraint is binding. Thus, in a scenario where a given country is only utilizing parts of the allocated CO_2_ target, an abatement cost of 0 will be obtained.

In Figure 3B, it is seen that a large number of the model countries have CO_2_ abatement costs of zero, indicating that these countries are not fully exploiting their assigned national emission targets. Considering the group of countries, which always utilize their allocated emissions (see Figure S1), and their incurred CO_2_ abatement costs shown in Figure 3B, these countries are seen to always have non-zero abatement costs. The abatement costs for these countries are ranging from 30 to 40 € per ton CO_2_ in most configurations of national reduction target allocations, but with outliers ranging much higher. The Efficiency schemes are seen as having a homogeneous CO_2_ abatement cost determined by the joint emissions reduction target. The change in global CO_2_ abatement costs between the Efficiency configurations with 55% and 70% reduction are reflecting the increased socio-economic burden associated with higher emissions reductions. The homogeneous CO_2_ abatement of the Efficiency scheme is associated with a very heterogeneous distribution of emission intensities. Contrary, in the Sovereignty configuration, CO_2_ reduction targets are assigned equally based on the national energy demand resulting in a rather homogeneous distribution of emission intensities. But, analyzing the CO_2_ abatement cost of the Sovereignty configuration reveals a heterogeneous distribution. This indicates that there is a trade-off to be made between a homogeneity in emission intensities and abatement costs. In the ability-to-pay approach, emissions are distributed inversely proportional to national gross domestic product GDP per capita. This redistribution of emissions with the ability-to-pay approach is discerned in Figure 3, where wealthy countries such as Germany and the Netherlands end up with low emissions while countries such as Romania, Macedonia, Poland, and Bulgaria feature higher emissions.

**Figure 4:**
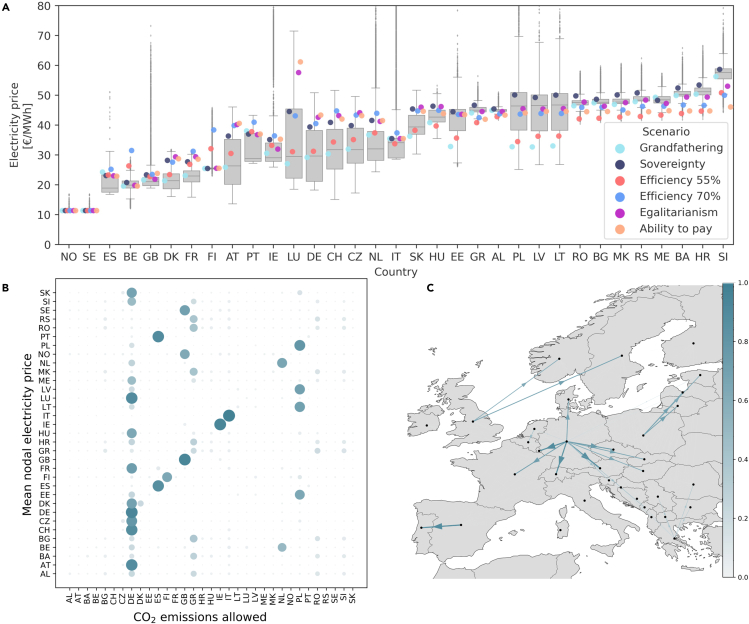
National mean electricity prices and the impact of emission targets (A) Shows a boxplot of mean national electricity prices for all model countries. In countries containing more than one power trading zone, the electricity price is calculated as a mean across all zones. (B and C) Shows the impact of national emission targets on the electricity price in all model countries. The impact factor is calculated as the Mean Decrease Impurity measure using the Extra Trees regressor. Impact strength is indicated with color and circle/arrow size. In panel (C) the arrow direction indicates the direction of the impact. For example, an arrow from Germany toward Austria indicates that Germany’s emission target has a large impact on Austria’s electricity prices.

The hourly national electricity price can be found as the Lagrange multiplier value of the national electricity balance constraints.^25^ Time-averaged electricity prices are shown in Figure 4A. Electricity prices are found to have a smaller spread for the individual countries compared to the abatement costs seen in Figure 3. The robustness of the electricity price does, however, depend on the country observed with countries at each end of the figure having more robust prices and countries toward the center having larger deviations. The countries observed to have constantly high prices are to a large extent the same countries that had high abatement costs. The observed electricity prices in Figure 4 span from 10 €/MWh to above 70 €/MWh for some outlier outcomes. This span in power prices is rather large compared to historic power prices which have been around 50 €/MWh for most European countries. Because so many different effort-sharing scenarios are modeled here, we can conclude that it is very unlikely that one can identify a scenario where high abatement costs and high electricity prices in these countries can be decoupled without additional political or economic measures.

Using the Extra-Trees regressor^26^^,^^27^ to estimate Sobol indices, the impact of national CO_2_ targets on the electricity price of other nations has been calculated. Figures 4B and 4C show the calculated impact factors. In Figure 4B each row in the matrix represents all impacts on the given country’s electricity price. Each row will sum to 1. The columns in the figure represent the impact a nation’s CO_2_ target has on the model countries. In Figure 4C impact strength is given by arrow size and color, and the impact direction is indicated by the arrowhead. E.g. the large arrow going from Germany to Austria indicates that the CO_2_ target of Germany has a large impact on the electricity price of Austria. By studying Figures 4B and 4C, it can be observed how large nations can have a significant impact on the electricity prices in surrounding nations. Germany is found to impact electricity prices in most central European countries, Poland has a large impact on the Baltic, and Greece has a large impact on the Balkans. Electricity prices are determined by supply and demand, as well as the marginal cost of producing electricity. When tight national CO_2_ targets are imposed on large nations, their desire to import electricity is likely to increase as a means of replacing fossil fuel-generated electricity. This increases demand in the surrounding nations, thus resulting in elevated electricity prices.

**Figure 5:**
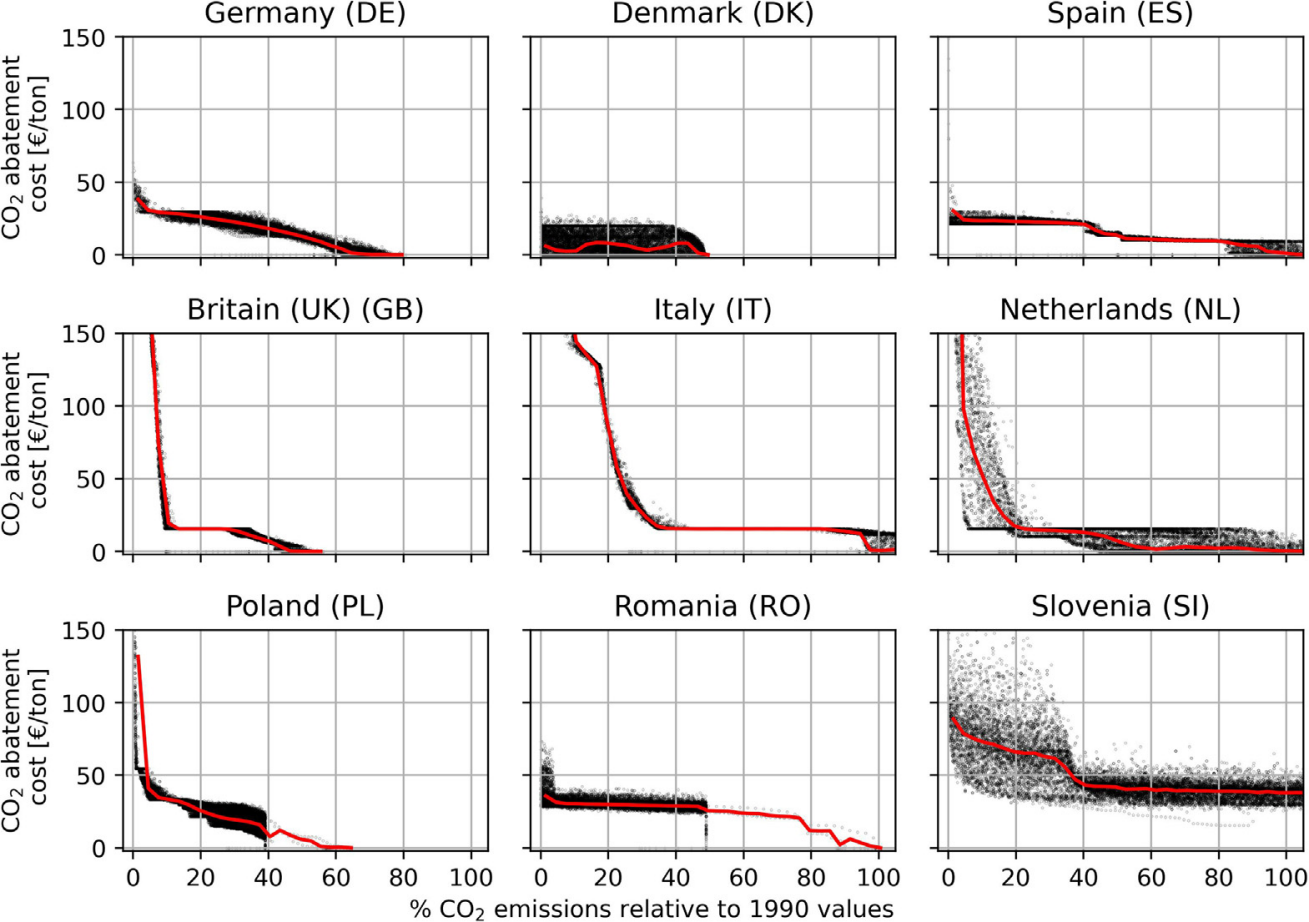
CO_2_ abatement cost dependence on emission level The figure shows CO_2_ abatement costs for a select group of model countries. The individual samples are shown with black dots and the mean with a red line. The figure is available for all model countries at Figure S3.

The price of abating CO_2_ emissions is naturally depending on the emission reduction level. Figure 5 shows the reliance of CO_2_ abatement cost on the emission reduction level for a select number of countries. The effect that other countries have on a specific abatement cost can be observed as the vertical spread of the point cloud. The MAA method used here ensures that the range is approximated well.

From the figure, it can be seen that CO_2_ abatement cost is tightly correlated with the emission reduction level in a highly non-linear manner. In the figure, some countries can be observed to have abatement costs increasing dramatically as their reduction levels approach zero. Analyzing the composition of the energy supply of these countries in Figures S4–S6, the countries where abatement costs increase drastically at low emissions can be observed to install large shares of renewables at these reduction levels. Thus, the increase in abatement cost is driven by a drastically increasing need for renewable energy sources. Drastic increases in the demand for renewable resources occur when flexible resources can no longer be provided by transmission from other countries as the transmission capacity is limited. The fluctuating mean line in Figure 5 for the case of Denmark (DK) shows that there is no direct relationship between the Danish emission reduction level and the Danish abatement costs. In a European context, Denmark is a small country, allowing the larger surrounding nations to have a large impact on their energy supply as observed in Figure 4. Simultaneously Denmark also has good availability of renewable resources thereby motivating the surrounding nations to exploit Denmark as an exporter of energy. The large impact of surrounding nations combined with the finite sample size of this study is what is causing the fluctuating mean abatement costs. Similar stories can be observed in Figure S3, for Lithuania (LT), Luxembourg (LU), and Latvia (LV). These are all small nations highly impacted by the actions of surrounding nations.

Studying how the five CO_2_ allocation approaches, from Table 1, are distributed in Figure S1, reveals that the Efficiency approach ensures that all national emission quotas are utilized 100%. In the Efficiency scheme, no country is assigned more emissions than needed, whereas the other allocation approaches result in inefficient allocations increasing total system cost. Especially the Grandfathering scheme leads to a large share of assigned emissions being left unused. It should be noted, that as only the electricity sector is considered in this research, the lower limit of 55% reductions provides a somewhat conservative benchmark, considering that the electricity sector is expected to be the first to be decarbonized. It is, however, important to note that the model is free to reach decarbonization levels above 55% and also does so. As several countries already have decarbonized their electricity sector to a high extent, they can be expected to find it economically optimal to avoid using all the emissions they are assigned. Norway, Sweden, and Switzerland can be seen to refrain from using their allocated emissions in most configurations of national reduction target allocations. This is following the findings shown in Figure 3.

## Discussion

The European power sector is in the early phase of a major transformation from a fossil-fuel-based system to relying mainly on renewable and low-carbon resources.^28^ However, efficient efforts to reduce emissions differ between countries that have different starting points, public preferences, geographical constraints, etc. Some countries have already installed high shares of carbon-neutral energy sources, while other countries are deeply reliant on fossil fuels.^29^ A historical example of the diversity among national strategies toward transforming their energy supply was seen in the response to the oil crisis in the 70s. Here vastly different strategies to reduce reliance on oil imports were employed. While Brazil took advantage of the low demand for locally produced sugarcane and started large-scale production of bio-ethanol, France invested heavily in nuclear infrastructure.^30^ The historical path-dependency in national energy supply not only sets the starting point for the green transition but can also breed an “energy culture” with a preference for specific technologies. Denmark, for instance, became a pioneer in wind power thanks to R&D efforts that began as early as the 1880s with Paul La Cour’s novel blade design.^31^

The results of this study show how European countries react very differently to changing national emission targets, revealing large inequality in the efforts required to decarbonize national electricity supplies. Where some nations find it cost-optimal to reduce emissions beyond the 55% ambition, other nations require a strong policy push to achieve the same goal. Under a global CO_2_ price—such as EU Emissions Trading System (ETS)—it will be cost-optimal to continue reliance on fossil fuel-based electricity production in countries with hard-to-decarbonize electricity supplies. If the inequality in decarbonization costs is not addressed, these countries will be left behind in the transformation of the European energy sector. Some of the countries where decarbonization of the electricity sector has been found especially costly include Slovenia, Greece, Poland, North Macedonia, Bulgaria, and Romania. Following the sovereignty effort-sharing principle would result in a more equitable distribution of CO_2_ intensity, but it will drive higher CO_2_ abatement costs in Poland and other countries with high emission intensities. The cost associated with supporting the green transition with financial support naturally entails a loss in national welfare as resources that could have otherwise been prioritized for other parts of society has to be used to reduce emissions from the energy sector. The highest abatement costs are found in nations with relatively low GDPs. Thus, if all European nations are expected to contribute equally, in terms of emission reductions, there is a potential for severely increased inequality among EU nations. Only through the strong financial support of the nations with hard-to-decarbonize electricity sectors can a just transition be achieved.

In this study, the national CO_2_ abatement costs associated with varying levels of national decarbonization have been identified. The CO_2_ abatement cost can be interpreted as the policy push required to obtain a given national emission reduction target. This policy push could, in practice, be implemented in several ways. Effective examples for incentivizing emission reductions include subsidies for renewable technologies, publicly funded projects, and performance standards.^32^ In this study, the magnitude of the policy push is determined but the specific policy tool is not discussed.

An overlap can be observed between the group of countries experiencing high abatement costs and the countries experiencing high electricity prices. Countries included in both groups include several Eastern European and Balkan countries. As these countries also tend to have high-income inequality, increasing electricity prices may significantly grow energy poverty. Thus, decarbonization governed solely by a global CO_2_ price may drastically increase social and economic inequality. This further increases the risk of inequality in the transition.

A fully economically efficient outcome is hard to obtain, as it requires close coordination and full information. Although in theory, EU ETS should ensure an efficient outcome; in practice, information asymmetries are distorting the clearing of markets. Moreover, there are sunk costs causing dirty power producers to continue operating, due to the differences between marginal and average costs of production. The results obtained in this work show how small deviations from the cost-optimal effort-sharing configuration will lead to increases in total system cost. The range of possible national CO_2_ reduction targets identified for the European electricity supply reveals that a cost increase of 5% from the cost-optimal solution is almost inevitable.

The differences in abatement costs per unit of carbon can be misleading as to the distributional consequences, as the abatement effort will be less demanding in countries that are already partly decarbonized such as France. Still, not only can unit costs be expected to be relatively high in Balkan countries, but these countries are also facing a greater transformation overall, involving high absolute costs. Thus, it is important to consider the volume of emissions that must be abated along with the marginal abatement cost.

A global price on CO_2_ emissions ensures economically efficient decarbonization of the European electricity sector but fails to provide a just energy transition. EU ETS is currently governing emission prices in all EU countries.^33^ The price of emission permits is the same across Europe, despite differences in GDP, income, and purchasing power. Countries with low incomes tend to have inefficient and dirty power sectors, further reinforcing the inequities, as the costs to shoulder decarbonization tend to be relatively larger for them. To ensure equity under EU ETS financial support must be provided to nations where CO_2_ abatement costs are highest. The commission proposes to create an ETS2 for transport and households; hence it is relevant to explore effort-sharing principles and their implications. Especially so as the Just Transition Fund^8^ is relatively loosely described.

### Limitations of the study

Weather and demand patterns are expected to change as a result of global warming and the general electrification of energy use. Investigating these effects is, however, beyond the scope of this paper. Only the electricity sector has been modeled. At a European level, the effects of sector coupling are only expected to be moderate by 2030, thus, this simplification is believed to provide only a minor source of error. If sector coupling was implemented, the electricity sector could be expected to achieve a higher decarbonization rate than involved with the 55% target, as it is considered easier to achieve here than in other sectors.

## STAR★Methods

### Key resources table

**Table:** 

REAGENT or RESOURCE	SOURCE	IDENTIFIER
**Deposited data**		

Zenodo: 30.000 ways go beyond 55p decarbonization of the European electricity sector	This paper	https://doi.org/10.5281/zenodo.7780611

**Software and algorithms**		

PyPSA: Python for Power System Analysis	PyPSA Developers 2015-2023	https://doi.org/10.5334/jors.188
PyPSA-Eur-Sec	Victoria et al.^34^	https://doi.org/10.1016/j.joule.2022.04.016

### Resource availability

#### Lead contact

Gorm Andresen, Email: gba@mpe.au.dk.

#### Materials availability

No new unique materials was generated in this study.

### Method details

In this section, the methods applied in this work will be thoroughly explained. The section starts with a general introduction and motivation for the choice of methods. This is followed by a detailed explanation of the energy system model and sampling method respectively.

In this research, an implementation of the MAA method^22^ using the Adaptive Metropolis-Hastings (AMH) sampler, an efficient implementation of an Markov chain Monte Carlo (MCMC) method,^35^ has been employed in combination with the PyPSA-Eur-Sec^23^ techno-economic optimization model of the European electricity supply system. The MAA method is used to identify and study 30.000 configurations of national emission targets under which the EU can reach its climate targets as a whole. A requirement of near-optimality is enforced for the configurations demanding that the increase in total system cost relative to the cost-optimal solution is below 18%. This is combined with a requirement of achieving emission reductions of at least 55% relative to 1990 values.

The constraint on near-optimality is based on the principles from Modeling to Generate Alternatives (MGA) where economically near-optimal model solutions are studied.^22^^,^^36^^,^^37^ The maximum allowable increase in system cost of 18% is chosen to allow for scenarios with a CO_2_ reduction upwards of 75% compared to the 55% reduction of the reference scenario. In a study by Trutnevyte et. al. the historic cost deviations from cost-optimality are found to be between 9-23% in the UK power system in the period from 1990 to 2014.^38^ In the results (see Figure 2), we observe that the peak in the distribution of scenarios is well below the 18% cut-off. This indicates that while the cut-off does rule out some scenarios, it does not significantly affect our results. A higher cut-off would simply extend the tail of the distribution to a higher cost, but the shape of the distribution below the cut-off would remain unchanged due to our uniform sampling method. Thus, the exact value of allowable cost increase is not critical, as long the majority of the underlying distribution is captured.

The European Green Deal^39^ sets a target to reduce European emissions by 55% compared to 1990 values by 2030. The 55% reductions target in the European Green Deal considers all sectors, and the electricity sector is expected to achieve higher emission reduction as it is generally believed to be the sector easiest to decarbonize.^40^ Therefore, the 55% target has been used as a lower bound and reference point in this work to study solutions achieving at least this goal, but mainly higher reduction levels. In addition to the requirements on system cost and emission reduction, all scenarios are further required to be capable of resulting in a technically feasible model solution, and National emissions must remain below the equivalent of supplying 150% of energy demand with coal. All requirements are listed in Table Feasibility requirements.

**Table Feasibility requirements. CO2 configurations scheme feasibility criteria**

a) The joint CO_2_ reductions must be equal to or greater than 55% relative to 1990.
b) Total system cost of the configuration of national reductions should not exceed the costoptimal scenario of 18%.
c) A technically feasible solution to the model exists.
d) National emissions must remain below the equivalent of supplying 150% of energy demand with coal.

#### Energy system optimization model

In this work, the joint capacity and dispatch energy system optimization model PyPSA-Eur-Sec model^23^ is used. The PyPSA-Eur-Sec model depends on data imports from the PyPSA-Eur model.^41^ The model formulated in this work represents a 2030 brownfield scenario of the European electricity supply spanning 33 ENTSO-E member countries, i.e. the model includes EU-27 without Cyprus and Malta, instead including Norway, Switzerland, Serbia, Bosnia-Herzegovina, Albania, Montenegro, Macedonia and, United Kingdom. The model is capable of optimizing investment and dispatch of technologies for the European electricity supply.

A brownfield scenario is generated where existing capacities that are planned to be in operation by 2030 are included in the model. The included brownfield capacities are seen in Figure 1 and Table S3. Existing conventional capacities are found in the power plant matching database,^42^ while renewable capacities are found in the IRENA annual statistics.^43^ The energy-generating technologies included are hydro, onshore wind, offshore wind, solar photovoltaic PV, combined cycle gas turbine (CCGT), open cycle gas turbine (OCGT), coal, lignite, nuclear, and oil. Furthermore, two storage technologies are included. These are hydrogen and battery storage. The technical parameters are listed in Supplemental 2. All existing plus the planned transmission capacities in the Ten Year Network Development Plan (TYNDP)^44^ are included. Transmission capacities are seen in Figure 1. Transmission capacities are clustered to one node per synchronous zone setup, with the nodes connected by representative high-voltage AC and DC lines.

The model can expand Technology capacities to meet energy demand. The cost of the expandable technologies is given in Table S2. Efficiency and emission data are available in Table S1. Technology costs are primarily based on the 2030 cost prediction given by the Danish Energy Agency in their technology data catalog.^45^ A discount rate of 7% has been used to calculate annualized costs using the annuity factor given in Equation 1. Here *r* is the discount rate and *n* is the technology lifetime.

(Equation 1) $$a=\frac{1-{\left(1+r\right)}^{-n}}{r}$$

Using one year of energy demand and weather data resolved in 3-hour time steps, the model determines the cost-optimal dispatch, power flows, and investment in new generator capacity. Transmission and generator capacities have been aggregated to one node per power synchronous zone, using the module created by Hörsch et al.^46^ The spatial and temporal resolution has been found to have a large impact on the results produced by energy system optimization models.^47^^,^^48^ In the trade-off between model precision and solving time, a choice of 3-hour time-steps and one node per synchronous zone is chosen, as it allows for a relatively fast solving time of the model while keeping errors within a tolerable range.^47^

The model of the European power sector is formulated as a linear optimization problem, consisting of an objective function along with a set of constraints. Throughout this description of the model, the model variables are split into two vectors namely **x** and **y**. Where **x** describes the national CO_2_ reduction target given by the AMH sampler **x** = *r*_*n*_ ∀ *n*. Here *r*_*n*_ is the national CO_2_ target in tons CO_2_ for all model countries *n*. The remaining variables **y** represent technology capacities and dispatch **y** = {**g**_*n,s,t*_*,***G**_*n,s*_*,***F**_*l*_}. Here index *s* is indexing the technology for all technologies included in the model, index *t* is indexing the hour for all hours in the year, and *l* represents the transmission line. The variables determined in the optimization process are thus:

• **g**_*n,s,t*_: Hourly dispatch of energy from the given plants in the given countries with the marginal cost **o***n,s.*
• **G**_*n,s*_: Total installed capacity of the given technologies in the given countries with the capital cost **c**_*n,s*_*.*
• **F**_*l*_: Total installed transmission capacity for all lines with the fixed annualized capacity cost **c**_*l*_*.*

The model is then formulated as a linear problem following the standard formulation given:

(Equation 2) $$\begin{array}{ccc} minimize & f_{0}\left(y\right) & \\ subject\;to & f_{i}\left(x,y\right)\leq 0 & i=1..m\\ & h_{i}\left(x,y\right)=0 & i=1..p \end{array}$$

The national CO_2_ targets **x** are given by the AMH sampler and are thus not optimized in the model.

Only the technical variables **y** are optimized in the optimization problem.

The objective function of the model is to minimize total system cost and can be formulated as follows:

(Equation 3) $$minimize\;f_{0}\left(x,y\right)=\sum_{n,s}c_{n,s}G_{n,s}+\sum_{l}c_{l}F_{l}+\sum_{n,s,t}o_{n,s}g_{n,s,t}$$

The model assumes perfect competition and foresight as well as long-term market equilibrium. For all model nodes and all hours in the year, a power balance constraint is enforced requiring that the energy demand **d**_*n,t*_ is fulfilled. Energy demand data is taken from the ENTSO-E data portal^49^ and decomposed into industrial and residential demand following the method given in.^41^ The incidence matrix describing the line connections is given by **K**_*n,l*_ and the hourly power flowing through each line is described as **f**_*l,t*_. The nodal power balance constraint can then be formulated as:

(Equation 4) $$\begin{array}{cc} \sum_{s}g_{n,s,t}-d_{n,t}-\sum_{l}K_{n,l}f_{l,t}=0 & \forall n,t \end{array}$$

The dispatch of each technology **g**_*n,s,t*_ is limited by the installed technology capacity **G**_*n,s*_. The dispatch of renewable energy generators such as wind and solar are furthermore limited by the hourly capacity factor **g**_*n,s,t*_. The capacity factor for conventional power plants is 1, whereas it is generated from weather data for renewable generators. A detailed explanation of the derivation of renewable generation potentials is given in.^41^

(Equation 5) $$0\leq g_{n,s,t}\leq {\bar{g}}_{n,s,t}G_{n,s}\;\forall n,s,t$$

Similarly, the power **f**_*l,t*_ flowing through the transmission lines is also limited by the installed capacity. As the direction of the transmission is without significance it is the absolute transmission |**f**_*l,t*_| that is limited.

(Equation 6) $$\left|f_{l,t}\right|\leq F_{l}\;\forall l,t$$

The maximum capacity allowed for each technology is determined by geographical potentials available $G_{n,s}^{\max}$.

(Equation 7) $$0\leq G_{n,s}\leq G_{n,s}^{\max}\;\;\forall n,s$$

CO_2_ emissions can be constrained in two ways. Either through a global constraint on emissions or by national constraints on emissions. The global CO_2_ reduction constraint is formulated as:

(Equation 8) $$\sum_{n,s,t}\frac{1}{\eta_{s}}g_{n,s,t}e_{s}-CAP_{CO_{2}}\leq 0$$

Here the *CAP*_*CO*2_ is the global emissions limit given in ton CO_2_. *η*_*s*_ is the generator efficiency and **e**_*s*_ is the CO_2_ equivalent emission intensity of the fuel. Note that only a single constraint is given here. A minimum requirement of 55% CO_2_ reductions has been used throughout this work, corresponding to an annual CO_2_ budget of 666.85 Mton CO_2_. Limiting emissions through national constraints can be done by defining a constraint for each country in the model.

(Equation 9) $$\sum_{s,t}\frac{1}{\eta_{s}}g_{n,s,t}e_{s}-r_{i}\leq 0$$

The national emissions targets **x** are given by the AMH sampler. The global CO_2_ constraint (Equation 8) is only used in the Efficiency scenario. In all other scenarios, the national CO_2_ targets are explicitly given, either by the sampler or following a certain allocation scheme using Equation 9.

The national CO_2_ reduction targets provided by the AMH sampler are included as constraints in the model, limiting CO_2_ emissions from energy generation in each of the modeled countries. Still, modeled countries are free to over-perform on the national CO_2_ reduction target if it is economically favorable.

Using the PyPSA-Eur-Sec model allows for a detailed investigation of the national cost of abating emissions. When the model is solved the Lagrange multipliers associated with every constraint are also obtained as an output. The value of these Lagrange multipliers represents the cost increase/decrease associated with tightening/loosening the constraint by one unit. By studying the Lagrange multiplier or also referred to as shadow price arising from enforcing national CO_2_ reduction constraints Equation 9. The shadow price of national CO_2_ reduction constraints can be interpreted as the marginal cost of abating further emissions in the given country and will be referred to as the CO_2_ abatement cost in this study. Similarly, the constraint on nodal power balance (Equation 4) results in a shadow price on power for each time step in the model.

This nodal power price produced can to a large extent be interpreted as a price replicating to spot markets.

#### Sampling method

To draw possible CO_2_ target configurations an implementation of the MAA method^22^ building on the Adaptive Metropolis-Hastings (AMH) sampler is implemented.^35^ The AMH sampler is based on a Markov Chain process where samples are continuously drawn from a proposal distribution centered around the previous sample point. By controlling the width of the proposal distribution continuously, the AMH sampler ensures efficient sampling. The AMH sampler is chosen as it is simple to implement while providing efficient sampling and fast mixing.^50^

An arbitrary CO_2_ target configuration can be denoted as the vector **x**, with each component of this vector *x*_*i*_ representing the national CO_2_ emission target of the *i*’th country relative to the total CO_2_ emission target.

The allowed emission for a given country can be determined as *x*_*i*_ ·*CO*2_*CAP*_, where the *CO*2_*CAP*_ is the total global amount of CO_2_ emissions allowed in tonnes of CO_2_. Realizations of the variables are denoted with subscript **x**_*t*_. It is important to note that the sum of **x** can be greater than 1, and thus the combined emission targets can add up to more than the total global amount of CO_2_ emissions allowed *CO*2_*CAP*_. As the rejection criteria are based on the realized emissions, a sample where the sum of **x** is greater than 1 can be accepted if not all allowed emissions are realized. Similarly **x** can also sum to less than 1. In such a case the global emission reductions will be less than the required 55% and the sample can be immediately rejected.

Given a starting point **x**_0_ the AMH sampler will continuously generate new sample proposals **x**′. New samples are drawn from the proposal distribution centered around the previous sample. The proposal distribution is defined as a uniform distribution around the previous sample point with the width *σ*. Thus the maximal change in each variable *x*_*i*_ per iteration is *σ/*2. There are however a few caveats. As the variables considered **x** are fractions of a total CO_2_ budget, they are constrained to be between 0 and 1. Therefore, the uniform distribution is bounded not to exceed this area. The starting point **x**_0_ used is the cost-optimal scenario also denoted as the Efficiency scenario. A burn-in period of 100 samples is used by discarding the first 100 samples of each chain to remove any bias toward the starting point.

(Equation 10) $$x^{\prime }∼U\left[\max\left(x_{t-1}-\frac{\sigma }{2},0\right),\min\left(x_{t-1}+\frac{\sigma }{2},1\right)\right]$$

The distribution width *σ* is tuned continuously as more information about the solutions space is obtained. By setting *σ* too low, the sampler will need an excessive amount of samples to explore the entire solution space. On the other hand, setting *σ* too high will result in the rejection of too many samples. By continuously monitoring the acceptance rate, it is possible to determine if the chain is taking either too short or long steps. If the acceptance rate is very high *σ* should be increased, and if the acceptance rate is low *σ* should be decreased. In practice, this is implemented by letting the sampler run for several iterations and evaluating the acceptance rate in that batch of samples. In this implementation of the AMH sampler, *σ* is updated by continuously monitoring the acceptance ratio of the samples. When the acceptance ratio is below a userspecified value, *σ* is incremented by a small amount *ε*, and vice versa when the acceptance ratio is too high. An *ε* value of 0.05 and a desired acceptance ratio of 80% have been used throughout this work.

The feasibility of a proposed sample **x**′ is evaluated using the energy system optimization model. If the solution to the energy system optimization model given **x**′ as input satisfies all criteria from Table Feasibility Requirements, the sample is accepted. Otherwise, the sample is rejected and a new proposal sample is drawn. When a proposed sample is accepted it is assigned index *t*, such that **x**_*t*_ = **x**′. If a sample is rejected the previous sample point is stored instead **x**_*t*_ = **x**_*t*−1_. The process of drawing samples from the proposal distribution and either accepting or rejecting them is repeated until sufficient sample size is reached.

The result is a set of **x** realizations that can ensure feasible operation of the model, global emission reductions higher than the base scenario, and a total system cost of no more than 18% higher than that of the base scenario. If enough samples are drawn the distribution of the set of realizations will approximate all solutions satisfying the above-mentioned criteria.

In practice, the above algorithm is implemented as a parallel process with multiple chains running simultaneously. The samples from the parallel chains can then be merged at the end of the sampling process.

#### Principle effort sharing schemes

Five principles for the allocation of national reduction targets have been used as references. Inspired by Zhou et al.,^16^ these are grandfathering, sovereignty, efficiency, egalitarianism, and ability to pay. The procedures for the allocation of national reduction targets, and conversely the emissions, for each of these five principles, are shown in Table 1. Using these five principles, six emission reduction configurations have been created as seen in Figure S7. The Efficiency 55% and Efficiency 70%, configurations correspond to using EU ETS at 55 and 70% joint reductions respectively, whereas, the Grandfathering, Sovereignty, Egalitarianism and, Ability to pay configurations represent alternatives to EU ETS. These configuration principles are implemented such that they all distribute the same CO_2_ budget, except for the efficiency 70% reduction configuration. As allowable emissions are left unused by some countries, as they find it economically favorable to do so, the total realized CO_2_ reduction for all configuration principles other than Efficiency, will be higher than the minimum goal of 55%. The configuration principles could alternatively be implemented to all have realized emissions corresponding to a 55% CO_2_ reduction. A choice was made to use configurations with equal CO_2_ budgets rather than equal realized emissions, as they represent a more diverse set of scenarios and better represent a decision process where budgets are allocated as national targets that will be realized, e.g. a decade later. For results using configurations with equal realized emissions see Supplemental 3.

#### Calculating impact factor

Using the ExtraTrees regression technique for non-linear regression, the Mean Decrease Impurity measure can be calculated.^26^^,^^27^ The value of the Mean Decrease Impurity measure can be interpreted as an equivalent to the total order of Sobol-indices. The implementations use the Scikit-learn: Machine Learning in Python package.^51^ The calculated indices provide a measure of the impact a given input variable has on the regressor output.

## Data Availability

• All original code and results have been deposited at Zenodo repository https://doi.org/10.5281/zenodo.7780611. • The software is under the Creative Commons IGO 3.0 license. • Any additional information required to reanalyze the data reported in this paper is available from the lead contact upon request.
