# Supplementary material for: Using Modeling All Alternatives to explore 55% decarbonization scenarios of the European electricity sector
Source: iScience. 2023 Apr 18;26(5):106677. doi: 10.1016/j.isci.2023.106677 (PMC10165455; doi:10.1016/j.isci.2023.106677)
Supplement: Document S1. Figures S1–S7 and Tables S1–S3 [file mmc1.pdf]

iScience, Volume *26*

Supplemental information

Using Modeling All Alternatives to explore 55% decarbonization scenarios of the European electricity sector

Tim T. Pedersen, Mikael Skou Andersen, Marta Victoria, and Gorm B. Andresen

Supplemental material for: Using Modeling All Alternatives to explore 55% decarbonization scenarios of the European electricity sector

Tim T. Pedersen [1,3,*], Mikael Skou Andersen[2,1], Marta Victoria[1,3], Gorm B. Andresen [1,3,**]

Supplemental 1. Unused emissions

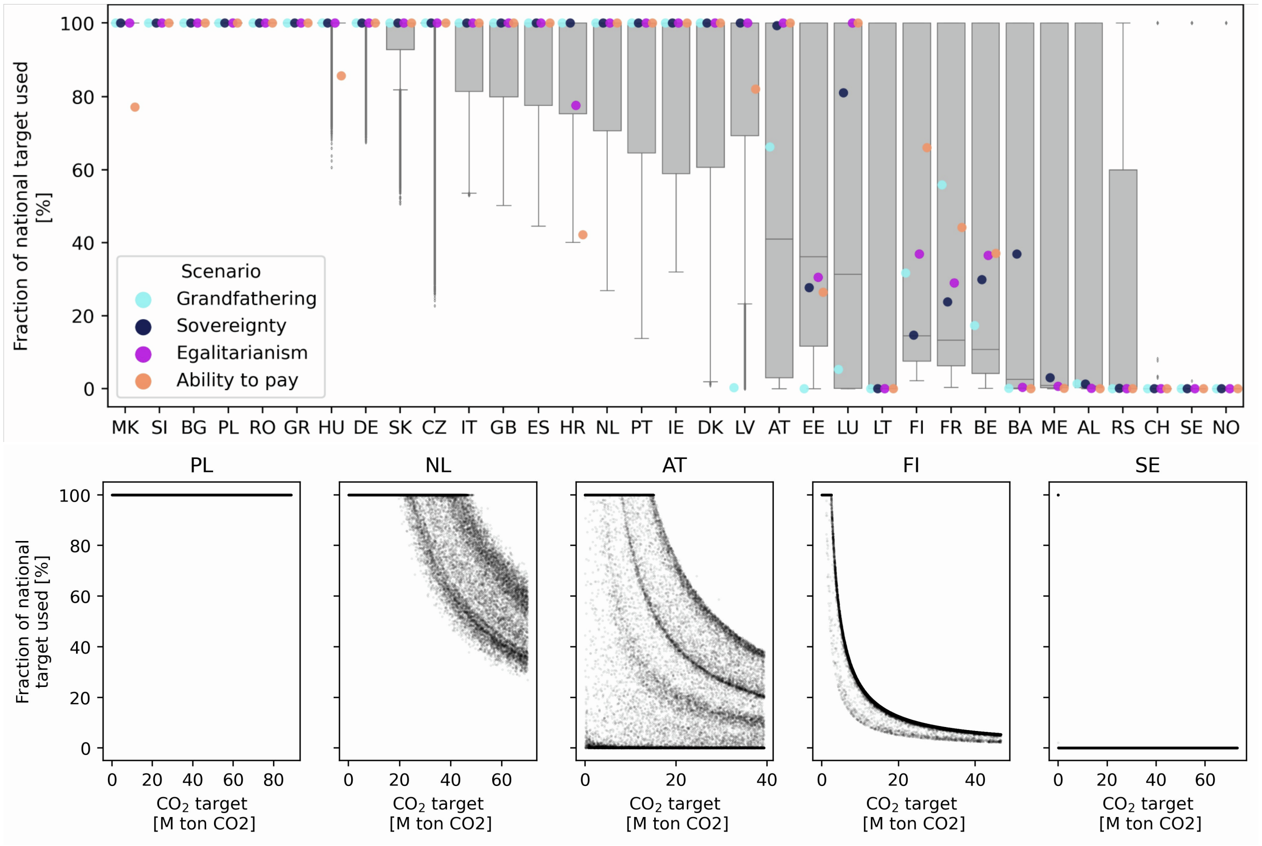

Figure S1: **Utilization of national emission targets** Related to Figure 3. a) A box plot of the utilization of the national reduction targets for the individual countries across all scenarios. A value of 100% means that the country is emitting as much CO_2 as their reduction target allows, whereas 0% indicates that the country has no emissions although the CO_2 reduction target is not 0. The reduction configurations Efficiency 55% and Efficiency 70% are not included on the figure as they per definition utilize all assigned emissions. b) Example countries. Reduction target utilization plotted against the total amount of target emissions.

Supplemental 2. Model assumptions

Table S1: **Technology data** Related to Figure 1. Emissions are given as t CO_2 per MWh electricity produced.

Technology	Efficiency<br>%	Emissions<br>ton CO_2/MWhe
OCGT	41	0.49
CCGT	58	0.34
Coal plant	33	1.00
Lignite plant	33	1.24
Oil plant	35	0.77
Electrolysis	66	0
Fuel Cell	50	0
Battery inverter	96	0

Table S2: **Technology costs of new technologies** Related to Figure 1

Technology	Capital cost	FOM	VOM	Lifetime
	Eur/kW	%/year	Eur/MWh	years
OCGT	435.2	1.78	4.5	25
Offshore wind turbine	1573.2	2.29	2.67	30
Offshore wind AC connection submarine	2685.0*	0	0	30
Offshore wind AC connection underground	1342.0*	0	0	30
Offshore wind AC station	250.0	0	0	30
Offshore wind DC connection submarine	2000.0*	0	0	30
Offshore wind DC connection underground	1000.0*	0	0	30
Offshore wind DC station	400	0	0	30
Onshore wind	1035.6	1.22	1.35	30
Utility scale solar PV	376.3	1.93	0	40
Electrolysis	550.0	5.0	0	25
Fuel Cell	1100.0	5.0	0	10
Hydrogen storage tank	44.0**	1.11	0	30
Hydrogen underground storage	2.0**	0	0	100
Battery inverter	160.0	0.34	0	25
Battery storage	142.0**	0	0	25

* Eur/MW/km

** Eur/kWh

Table S3: **Existing generator technology capacities by 2030 in MW** Related to Figure 1

	Offshore wind	Onshore wind	Run off river	Solar PV	CCGT	OCGT	Coal	Lignite	Nuclear	Oil
AT	0.0	3132.7	4478.5	1438.6	2481.7	1313.5	991.5	0.0	0.0	0.0
BA	0.0	50.6	0.0	0.0	0.0	0.0	0.0	0.0	0.0	0.0
BE	1185.9	2074.8	59.0	3984.5	3801.9	1460.6	1524.8	0.0	5925.8	0.0
BG	0.0	691.0	22.4	1029.0	0.0	782.0	4963.7	3993.0	2000.0	0.0
CH	0.0	63.0	5280.0	2171.0	0.0	0.0	0.0	0.0	3430.0	0.0
CZ	0.0	316.2	40.2	2074.3	336.8	0.0	7184.7	725.7	2660.0	0.0
DE	6396.0	52447.0	2997.0	45179.0	18120.9	8044.3	28069.4	20833.5	15788.4	3696.4
DK	1708.1	4431.2	0.0	991.0	100.0	1427.4	3629.9	0.0	0.0	665.0
EE	0.0	329.8	0.0	25.4	173.0	250.0	0.0	0.0	0.0	2111.0
ES	0.0	23433.1	16.4	4753.5	24344.3	2942.6	6519.7	3081.2	7572.6	3533.4
FI	67.0	1971.3	1289.6	123.0	648.0	677.7	3039.7	0.0	2784.0	1225.4
FR	0.0	14898.1	5780.8	9604.0	5611.0	1066.0	4293.3	0.0	63130.0	7172.1
GB	8212.7	13553.9	685.2	13107.3	32824.3	921.5	14475.0	0.0	11261.0	2801.9
GR	0.0	2877.5	103.1	2650.6	4482.0	417.0	1550.0	3905.0	0.0	0.0
HR	0.0	580.3	278.7	67.4	369.6	82.5	304.3	0.0	0.0	647.8
HU	0.0	335.0	19.7	724.0	1259.2	2368.7	42.3	1180.2	1886.8	410.0
IE	25.2	3650.9	216.0	21.8	2946.0	1320.0	855.0	0.0	0.0	907.0
IT	0.0	10230.2	6563.7	20073.6	34438.1	6491.8	10926.5	0.0	0.0	6145.0
LT	0.0	532.0	0.0	81.9	0.0	1575.0	0.0	0.0	0.0	0.0
LU	0.0	114.2	30.9	124.7	350.5	0.0	0.0	0.0	0.0	0.0
LV	0.0	62.9	642.1	0.0	1025.0	0.0	0.0	0.0	0.0	0.0
ME	0.0	118.0	0.0	0.0	0.0	0.0	0.0	0.0	0.0	0.0
MK	0.0	37.0	41.6	17.0	0.0	0.0	0.0	824.0	0.0	0.0
NL	957.0	3491.0	0.0	4522.0	13582.0	3991.0	5591.0	0.0	492.0	0.0
NO	0.0	1708.0	0.0	53.4	450.0	773.1	0.0	0.0	0.0	0.0
PL	0.0	5762.1	14.4	562.0	326.0	1032.9	21588.5	9406.0	0.0	345.0
PT	0.0	5172.4	1615.5	665.4	3829.0	0.0	1756.0	0.0	0.0	0.0
RO	0.0	3243.0	870.4	1385.7	1080.0	2282.0	1506.0	4779.2	1298.0	87.5
RS	0.0	25.0	0.0	0.0	0.0	0.0	0.0	0.0	0.0	0.0
SE	204.0	7097.0	1955.9	481.0	708.0	0.0	130.0	0.0	9532.0	2135.0
SI	0.0	0.0	861.3	251.8	832.0	449.0	246.0	944.0	727.0	143.6
SK	0.0	0.0	641.3	533.0	648.0	0.0	440.0	486.0	1940.0	0.0

Supplemental 3. Scenarios with equal realized emissions

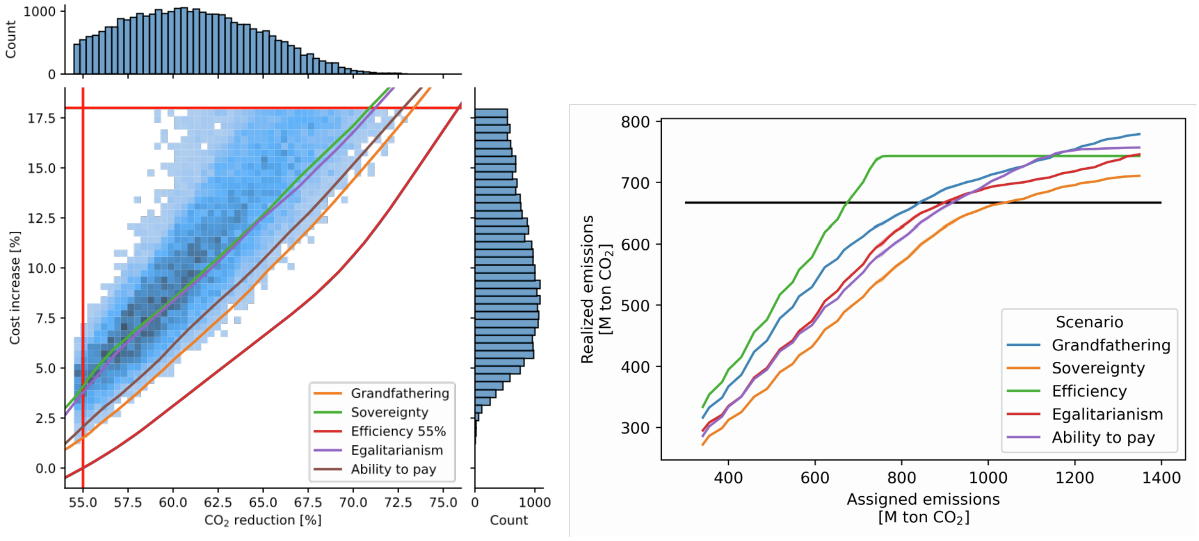

Figure S2: **Swept scenarios** A supplement to Figure 2. a) Shows the relationship between cost increase and CO_2 reduction for all configuration strategies. b) Realized CO_2 emissions from all configuration strategies plotted against the sum of assigned CO_2 targets. The horizontal black line represents the CO_2 budget associated with a 55% CO_2 reduction.

Supplemental 4. Detailed model results

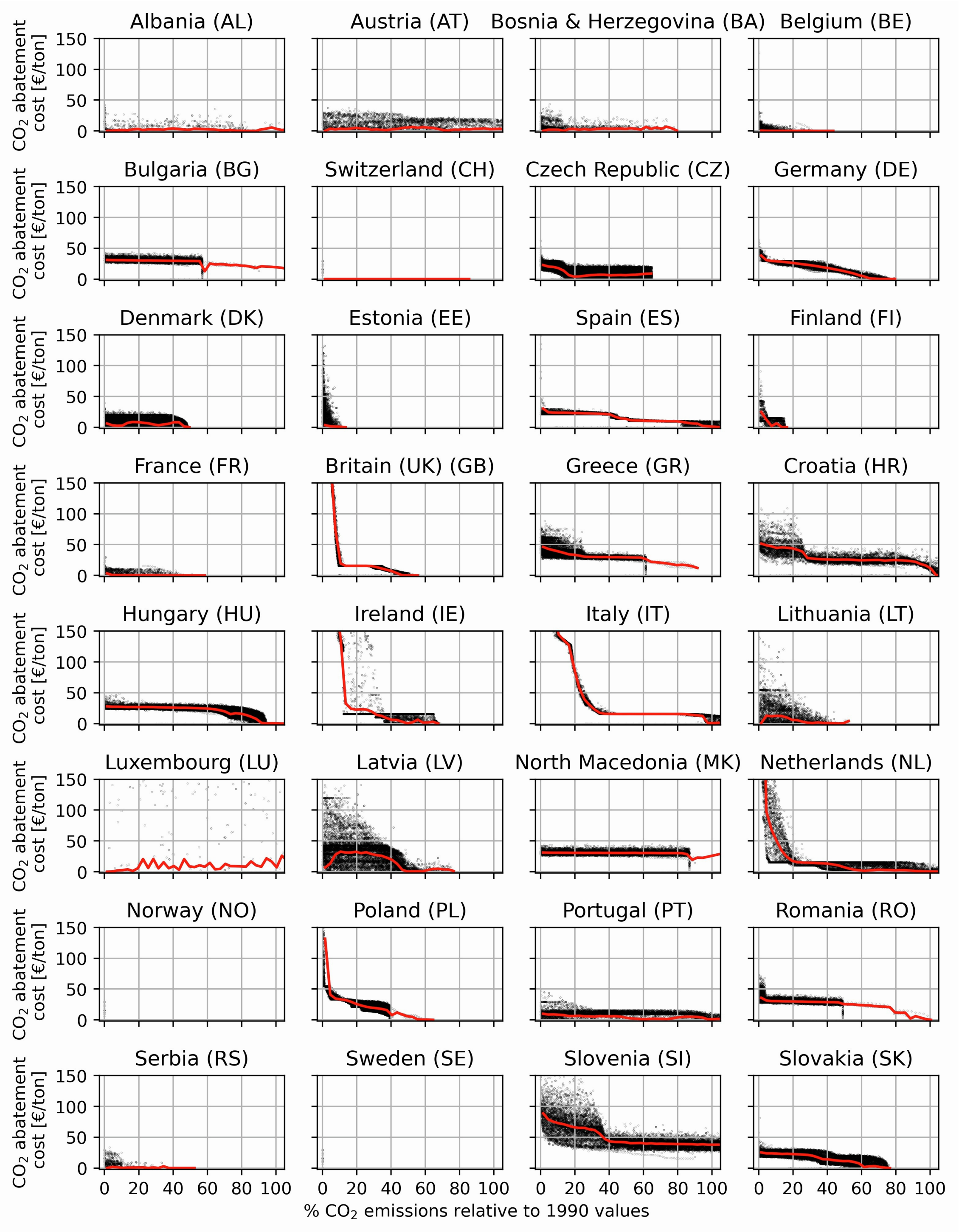

Figure S3: **CO_2 abatement cost** Related to Figure 5. CO_2 abatement cost for all model countries plotted against the CO_2 reduction level relative to 1990 values. Sample points are shown with black dots and the sample mean is shown with a red line.

1990 emission values are not available for Montenegro (ME) and the country has therefore been excluded from the figure.

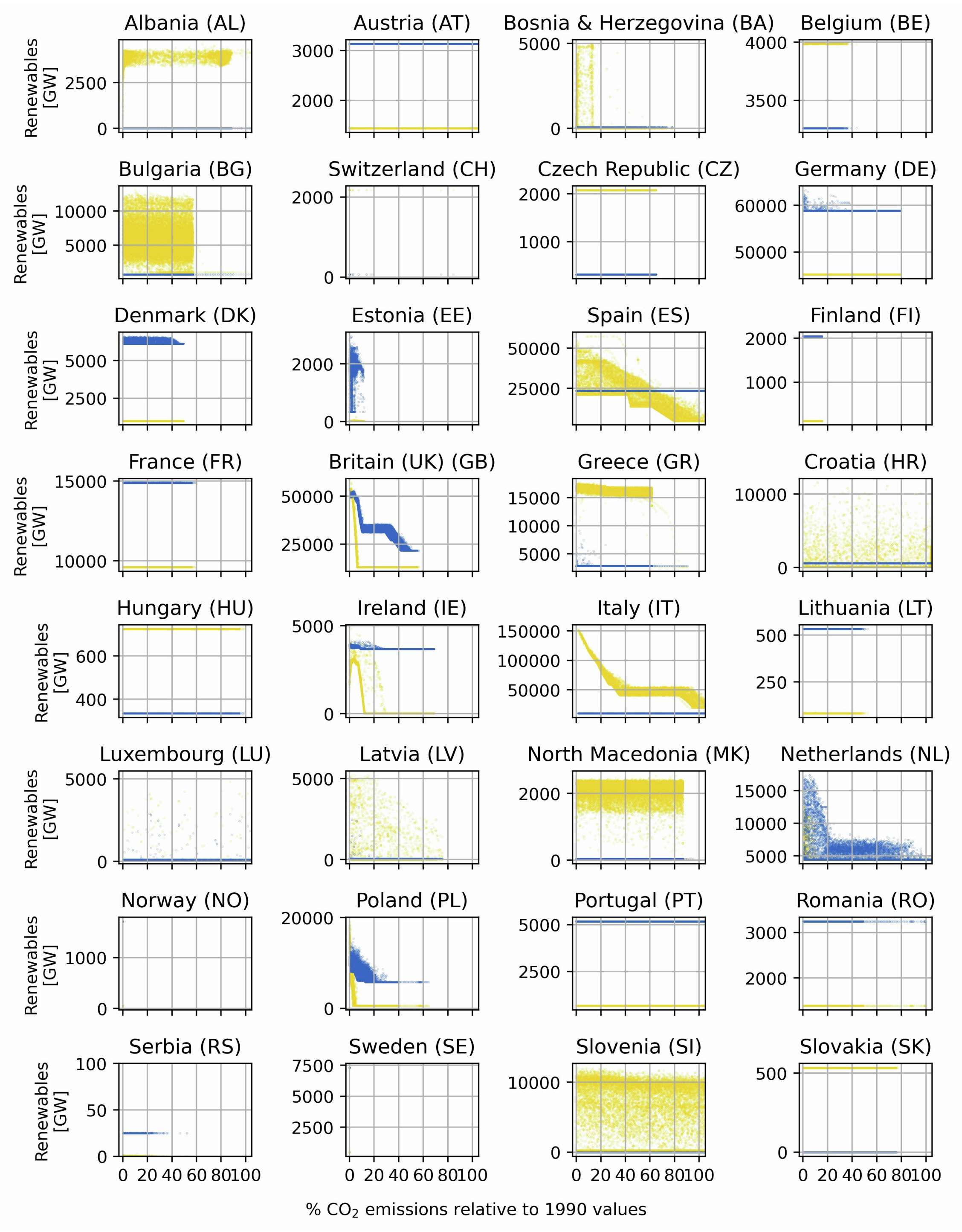

Figure S4: **Renewable energy generator capacities** Related to Figure 1. Renewable energy generator capacities plotted against CO_2 reduction levels. Every sample is shown as a single dot. Solar PV capacity is indicated by yellow dots, and wind turbine capacity with blue dots. 1990 emission values are not available for Montenegro (ME) and the country has therefore been excluded from the figure.

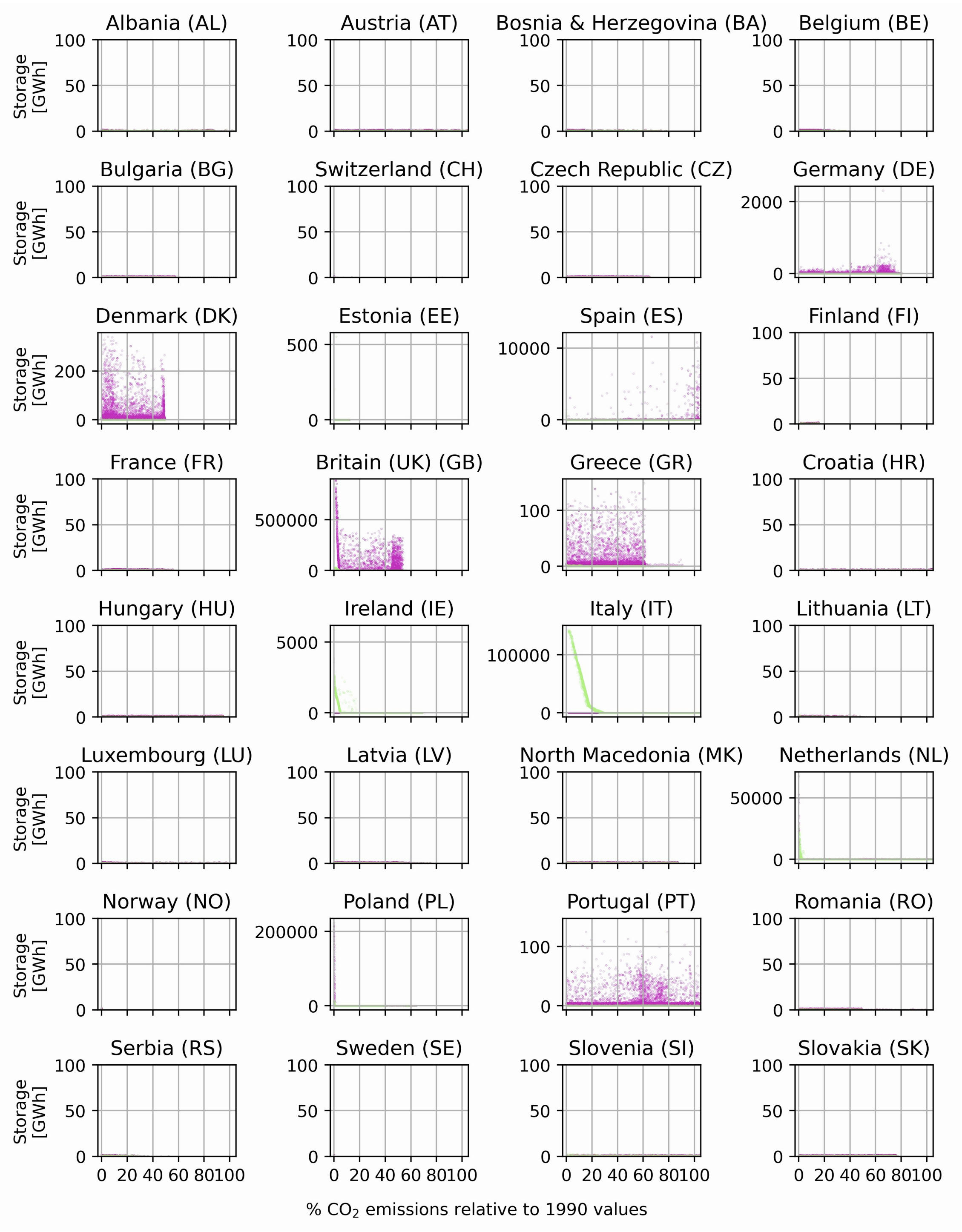

Figure S5: **Storage capacity for all model countries measured** Related to Figure 1. Storage capacity for all model countries measured in GWh storage capacity. Every sample is shown as a single dot. Battery storage is indicated by green dots, while H2 storage is shown with purple. 1990 emission values are not available for Montenegro (ME) and the country has therefore been excluded from the figure.

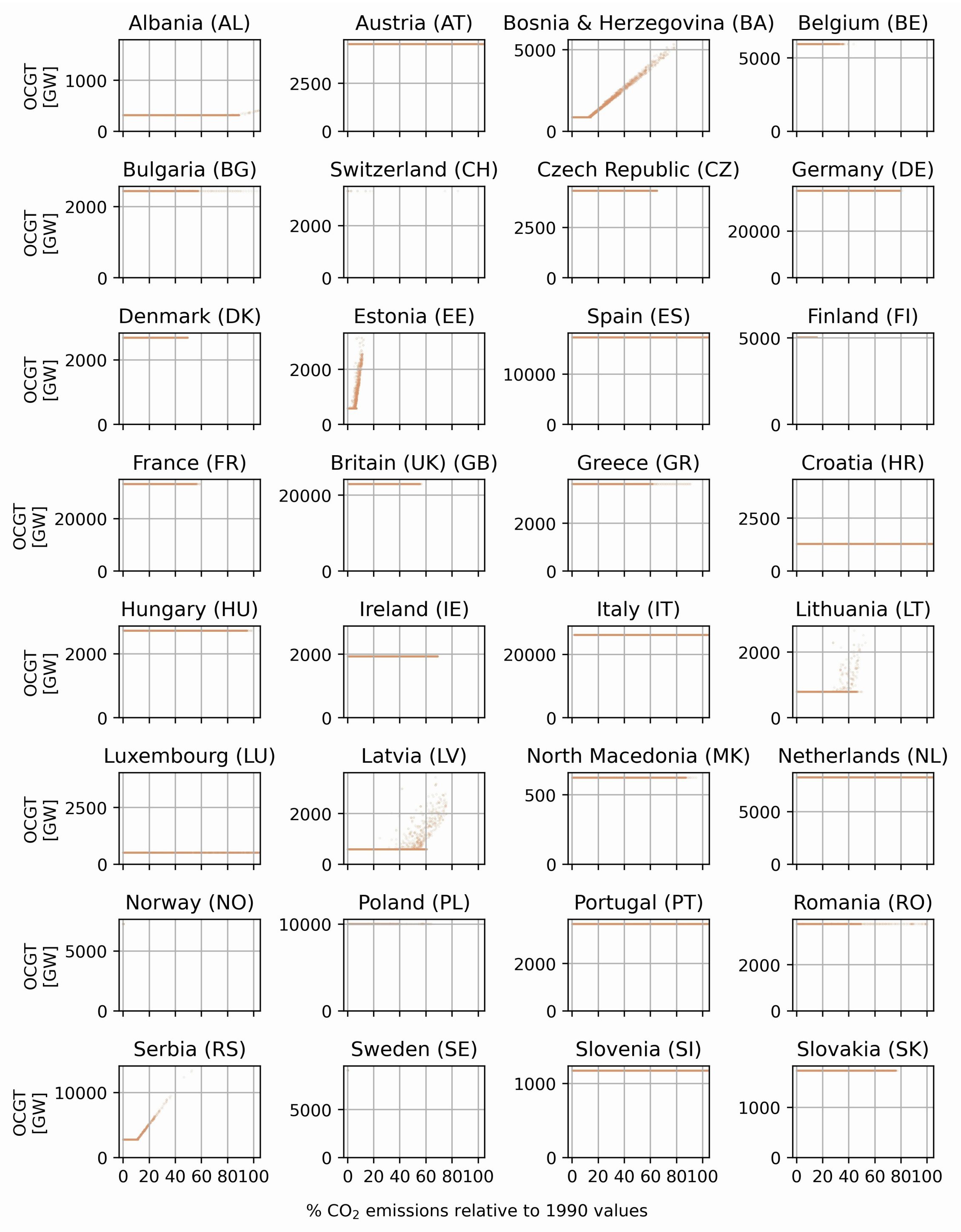

Figure S6: **Open cycle gas turbine (OCGT) capacity** Related to Figure 1. Open cycle gas turbine (OCGT) capacity for all model countries. Every sample is shown as a single dot. OCGT is the only extendable non-renewable energy source included in the model. 1990 emission values not available for Montenegro (ME) and the country has therefore been excluded from the figure.

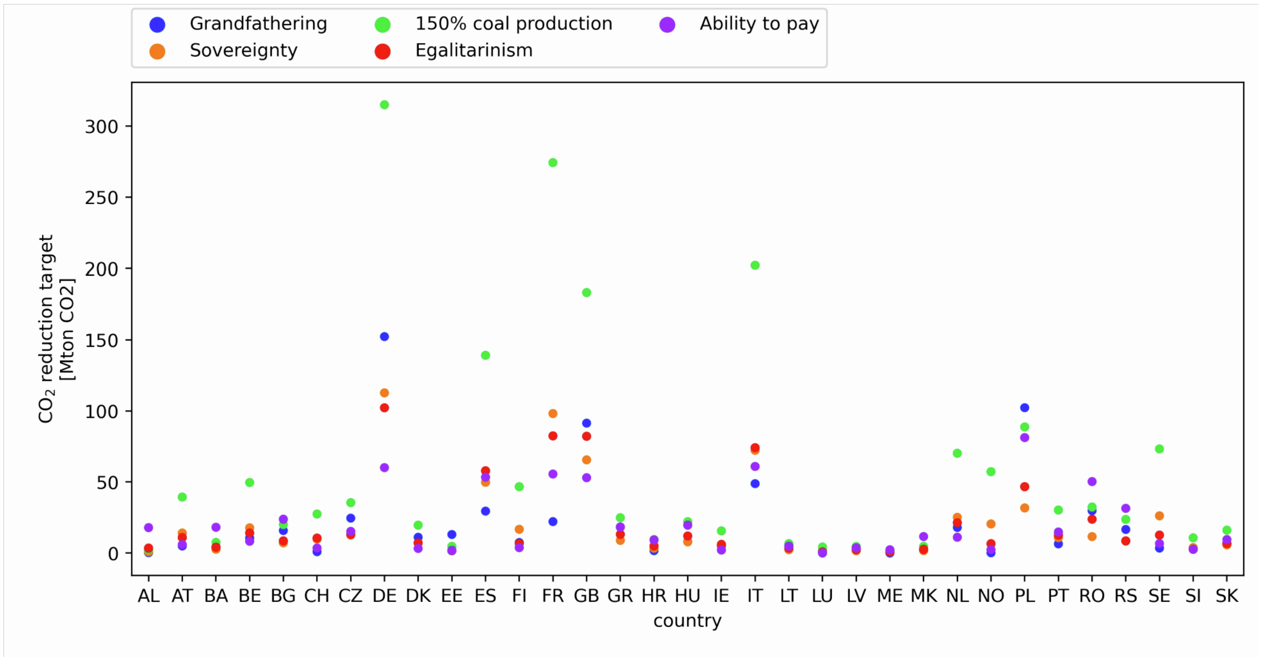

Figure S7: **CO2 target layouts for the scenarios used** Related to Figure 2. CO2 target layouts for the scenarios used and the 150% coal production upper limit. The 150% coal upper limit is calculated as a load of each nation multiplied by an emission factor of 0.45[tCO_2 per MWh] times 1.5.

References

[1] W. Nordhaus. (2019). Climate change: The ultimate challenge for economics. American Economic Review 109 (6) 1991–2014. doi:DOI:10.1257/aer.109.6.1991.

[2] European Council. Communication from the commission - the european green deal, https://eur-lex.europa.eu/legal-content/EN/ALL/?uri=COM:2019:640:FIN

[3] Van den Berg, N. J., van Soest, H. L., Hof, A. F., den Elzen, M. G., van Vuuren, D. P., Chen, W., Drouet, L., Emmerling, J., Fujimori, S., H¨ohne, N., et al. (2020). Implications of various effort-sharing approaches for national carbon budgets and emission pathways. *Climatic Change*, 162(4):1805–1822.

[4] European Council. Nationally determined contributions under the paris agreement. synthesis report by the secretariat. https://unfccc.int/documents/306848

[5] Tol, R. S. (2009). The economic effects of climate change. *Journal of economic perspectives*, 23(2):29–51.

[6] Hardin, G. (1968). The tragedy of the commons: the population problem has no technical solution; it requires a fundamental extension in morality. *science*, 162(3859):1243–1248.

[7] Bauer, N., Bertram, C., Schultes, A., Klein, D., Luderer, G., Kriegler, E., Popp, A., and Edenhofer, O. (2020). Quantification of an efficiency–sovereignty trade-off in climate policy. *Nature*, 588(7837):261–266.

[8] European Commision. The just transition mechanism. https://ec.europa.eu/info/strategy/priorities-2019-2024/european-green-deal/finance-and-green-deal/just-transition-mechanism_en

[9] Jenkins, K., McCauley, D., Heffron, R., Stephan, H., and Rehner, R. (2016). Energy justice: a conceptual review. *Energy Research & Social Science*, 11:174–182.

[10] Maguire, D., & Shaw, C. (2021). Fair energy transition for all-literature review. Climate Outreach: Oxford, UK.

[11] Levenda, A., Behrsin, I., and Disano, F. (2021). Renewable energy for whom? a global systematic review of the environmental justice implications of renewable energy technologies. *Energy Research & Social Science*, 71:101837.

[12] Golubchikov, O. and O'Sullivan, K. (2020). Energy periphery: Uneven development and the precarious geographies of low-carbon transition. *Energy and Buildings*, 211:109818.

[13] Böhringer, C., Rutherford, T. F., and Tol, R. S. (2009). The eu 20/20/2020 targets: An overview of the emf22 assessment. *Energy economics*, 31:S268–S273. https://doi.org/10.1016/j.eneco.2009.10.010

[14] Grunewald, P. (2017). Renewable deployment: Model for a fairer distribution. *Nature Energy*, 2(9):1–2. [18] Haario, H., Saksman, E., Tamminen, J., et al. (2001). An adaptive metropolis algorithm. *Bernoulli*, 7(2):223–242.

[15] Sovacool, B. K. and Dworkin, M. H. (2015). Energy justice: Conceptual insights and practical applications. *Applied Energy*, 142:435–444.

[16] Zhou, P. and Wang, M. (2016). Carbon dioxide emissions allocation: A review. *Ecological Economics*, 125:47–59.

[17] Alcaraz, O., Buenestado, P., Escribano, B., Sureda, B., Turon, A., and Xercavins, J. (2018). Distributing the global carbon budget with climate justice criteria. *Climatic change*, 149(2):131–145.

[18] Markowitz, E. M. and Shariff, A. F. (2012). Climate change and moral judgement. *Nature Climate Change*, 2(4):243–247. doi:https://doi.org/10.1038/nclimate1378.

[19] Schwenk-Nebbe, L. J., Victoria, M., Andresen, G. B., and Greiner, M. (2020). Co2 quota attribution effects on the european electricity system comprised of self-centred actors. *SSRN Electronic Journal*. https://doi.org/10.2139/ssrn.3689207.

[20] Sasse, J.-P. and Trutnevyte, E. (2019). Distributional trade-offs between regionally equitable and costefficient allocation of renewable electricity generation. *Applied Energy*, 254:113724.

[21] Drechsler, M., Egerer, J., Lange, M., Masurowski, F., Meyerhoff, J., and Oehlmann, M. (2017). Efficient and equitable spatial allocation of renewable power plants at the country scale. *Nature Energy*, 2(9):1–9.

[22] Pedersen, T. T., Victoria, M., Rasmussen, M. G., and Andresen, G. B. (2021). Modeling all alternative solutions for highly renewable energy systems. *Energy*.

[23] Brown, T., martavp, lisazeyen, Maria, M., Leon, and Neumann, F. (2020). Pypsa/pypsa-eur-sec: Pypsaeur-sec version 0.4.0. doi:10.5281/zenodo.4317529.

[24] European Environmental Agency (2021). Greenhouse gas emission intensity of electricity generation in europe. https://www.eea.europa.eu/ims/greenhouse-gas-emission-intensity-of-1

[25] Priesmann, J., Nolting, L., and Praktiknjo, A. (2019). Are complex energy system models more accurate? an intra-model comparison of power system optimization models. *Applied Energy*, 255:113783.

[26] Jaxa-Rozen, M. and Kwakkel, J. (2018). Tree-based ensemble methods for sensitivity analysis of environmental models: A performance comparison with sobol and morris techniques. *Environmental Modelling & Software*, 107:245–266.

[27] Jaxa-Rozen, M., Pratiwi, A. S., and Trutnevyte, E. (2021). Variance-based global sensitivity analysis and beyond in life cycle assessment: an application to geothermal heating networks. *The International Journal of Life Cycle Assessment*, 26:1008–1026.

[28] Victoria, M., Zhu, K., Brown, T., Andresen, G. B., and Greiner, M. (2020). Early decarbonisation of the european energy system pays off. *Nature communications*, 11(1):1–9. https://doi.org/10.1038/ s41467-020-20015-4.

[29] Eurostat. Greenhouse gas emissions by source sector. https://ec.europa.eu/eurostat/web/products-datasets/-/sdg_13_10

[30] Solomon, B. D. and Krishna, K. (2011). The coming sustainable energy transition: History, strategies, and outlook. *Energy Policy*, 39(11):7422–7431.

[31] Dyrhauge, H. (2017). Denmark: a wind-powered forerunner. In *A guide to EU renewable energy policy*, pages 85–103. Edward Elgar Publishing.

[32] Newell, R. G. Federal climate policy 101: Reducing emissions. Resources for the Future. https://www.rff.org/publications/explainers/federal-climate-policy-101/

[33] European Commision, Eu emissions trading system (eu ets). https://ec.europa.eu/clima/policies/ets_en

[34] Haario, H., Saksman, E., & Tamminen, J. (2001). An adaptive Metropolis algorithm. *Bernoulli*, 223-242. https://doi.org/10.2307/3318737

[35] Brill Jr, E. D., Chang, S.-Y., and Hopkins, L. D. (1982). Modeling to generate alternatives: The hsj approach and an illustration using a problem in land use planning. *Management Science*, 28(3):221–235. https://doi.org/10.1287/mnsc.28.3.221.

[36] Neumann, F. and Brown, T. (2021). The near-optimal feasible space of a renewable power system model. *Electric Power Systems Research*, 190. https://doi.org/10.1016/j.epsr.2020.106690.

[37] Trutnevyte, E. (2016). Does cost optimization approximate the real-world energy transition? *Energy*, 106:182–193.

[38] European Union. 2030 climate target plan, https://ec.europa.eu/clima/policies/eu-climate-action/2030_ctp_en

[39] Victoria, M., Zhu, K., Brown, T., Andresen, G. B., and Greiner, M. (2019). The role of storage technologies throughout the decarbonisation of the sector-coupled european energy system. *Energy Conversion and Management*, 201:111977.

[40] Hörsch, J., Hofmann, F., Schlachtberger, D., and Brown, T. (2018). Pypsa-eur: An open optimisation model of the european transmission system. *Energy Strategy Reviews*, 22:207–215. https://doi.org/ 10.1016/j.esr.2018.08.012.

[41] Hofmann, F. and H¨orsch, J. (2019). Fresna - powerplantmatching v.0.4.1. doi:https://doi.org/10.5281/ zenodo.3358985.

[42] IRENA. Anual statistics. Technical report, IRENA. https://www.irena.org/Statistics/Download-Data

[43] Ento-E, Ten year network development plan. https://tyndp.entsoe.eu/tyndp2018/

[44] Danish Energy Agency. Technology data catalogues. https://ens.dk/en/our-services/projections-and-models/technology-data

[45] Hörsch, J. and Brown, T. (2017). The role of spatial scale in joint optimisations of generation and transmission for european highly renewable scenarios. In *2017 14th international conference on the European Energy Market (EEM)*, pages 1–7. IEEE.

[46] Schyska, B. U., Kies, A., Schlott, M., von Bremen, L., and Medjroubi, W. (2021). The sensitivity of power system expansion models. *Joule*, 5(10):2606–2624.

[47] Frysztacki, M. M., H¨orsch, J., Hagenmeyer, V., and Brown, T. (2021). The strong effect of network resolution on electricity system models with high shares of wind and solar. *Applied Energy*, 291:116726.

[48] ENSTO-E. Data portal. https://www.entsoe.eu/data/data-portal/consumption/

[49] Solonen, A., Ollinaho, P., Laine, M., Haario, H., Tamminen, J., and J¨arvinen, H. (2012). Efficient mcmc for climate model parameter estimation: Parallel adaptive chains and early rejection. *Bayesian Analysis*, 7(3):715–736.

[50] Pedregosa, F., Varoquaux, G., Gramfort, A., Michel, V., Thirion, B., Grisel, O., Blondel, M., Prettenhofer, P., Weiss, R., Dubourg, V., Vanderplas, J., Passos, A., Cournapeau, D., Brucher, M., Perrot,

M., and Duchesnay, E. (2011). Scikit-learn: Machine learning in Python. *Journal of Machine Learning Research*, 12:2825–2830.
